# Ventral pallidal regulation of motivated behaviors and reinforcement

**DOI:** 10.3389/fncir.2023.1086053

**Published:** 2023-02-02

**Authors:** Carina Soares-Cunha, Jasper A. Heinsbroek

**Affiliations:** ^1^Life and Health Sciences Research Institute (ICVS), School of Medicine, University of Minho, Braga, Portugal; ^2^ICVS/3B’s-PT Government Associate Laboratory, Braga/Guimarães, Portugal; ^3^Department of Anesthesiology, University of Colorado, Anschutz Medical Campus, Aurora, CO, United States

**Keywords:** ventral pallidum (VP), substance use disorder, motivation, reward, aversion

## Abstract

The interconnected nuclei of the ventral basal ganglia have long been identified as key regulators of motivated behavior, and dysfunction of this circuit is strongly implicated in mood and substance use disorders. The ventral pallidum (VP) is a central node of the ventral basal ganglia, and recent studies have revealed complex VP cellular heterogeneity and cell- and circuit-specific regulation of reward, aversion, motivation, and drug-seeking behaviors. Although the VP is canonically considered a relay and output structure for this circuit, emerging data indicate that the VP is a central hub in an extensive network for reward processing and the regulation of motivation that extends beyond classically defined basal ganglia borders. VP neurons respond temporally faster and show more advanced reward coding and prediction error processing than neurons in the upstream nucleus accumbens, and regulate the activity of the ventral mesencephalon dopamine system. This review will summarize recent findings in the literature and provide an update on the complex cellular heterogeneity and cell- and circuit-specific regulation of motivated behaviors and reinforcement by the VP with a specific focus on mood and substance use disorders. In addition, we will discuss mechanisms by which stress and drug exposure alter the functioning of the VP and produce susceptibility to neuropsychiatric disorders. Lastly, we will outline unanswered questions and identify future directions for studies necessary to further clarify the central role of VP neurons in the regulation of motivated behaviors.

**Significance:** Research in the last decade has revealed a complex cell- and circuit-specific role for the VP in reward processing and the regulation of motivated behaviors. Novel insights obtained using cell- and circuit-specific interrogation strategies have led to a major shift in our understanding of this region. Here, we provide a comprehensive review of the VP in which we integrate novel findings with the existing literature and highlight the emerging role of the VP as a linchpin of the neural systems that regulate motivation, reward, and aversion. In addition, we discuss the dysfunction of the VP in animal models of neuropsychiatric disorders.

## Part 1: Neuroanatomy of the ventral pallidum

A large body of research shows that the ventral pallidum (VP) is a critical regulator of adaptive and exploratory behaviors, motivated states, reward-based learning, and hedonic processing (Kretschmer, 2000; Root et al., 2015). The VP was originally proposed to be a limbic-motor interface through which emotional and motivational information directs movement (Heimer et al., 1982; Mogenson et al., 1993), but more recent work indicates a particularly prominent role for the VP in reward processing, reinforcement, and motivation (Smith et al., 2009; Root et al., 2015). As a central node in ventral basal ganglia circuits, the VP integrates emotional and reward-related information from the ventral striatum, and prominently regulates the activity of the ventral mesencephalon dopamine system. In addition, the VP regulates emotion and motivation through its interconnectivity with brain stem areas, the extended amygdala, and the hypothalamus (Tripathi et al., 2013). The VP also regulates attention, reward learning, and cognitive processes through direct projections to the amygdala and prefrontal cortex, and indirect inputs to the prefrontal cortex that are relayed by the mediodorsal thalamus (MD; Kalivas et al., 1999; Zaborszky et al., 2012). Work in the last decade has begun to dissect the nuanced contributions of genetically distinct populations of VP neurons and subcircuits to motivated behaviors. In addition, recent work is beginning to disentangle the complex roles of striatopallidal and non-basal ganglia inputs to the VP (Root et al., 2015; Soares-Cunha et al., 2016a). Given its central role in the regulation of emotion, motivation, and reward processing, VP dysfunction is heavily implicated in neuropsychiatric disorders of motivation, including anxiety, depression, and substance use disorders (SUDs). This review will discuss recent advances in our understanding of VP cell- and subcircuit-specific contributions to motivated states and their dysregulation in animal models of depression, anxiety, and SUDs. First, the neuroanatomy and cellular heterogeneity of the VP will be discussed. Next, we will cover the main functional roles of the VP pertaining to reward processing, reinforcement, and motivated behaviors. Finally, recent insights into the cell- and subcircuit-specific regulation of VP afferents and efferents in reward processing and motivation will be covered (Supplementary Table 1), as well as changes in the functioning of these circuits produced by stress and drugs of abuse.

### Neuroanatomical connectivity

The VP was first described by Heimer and Wilson (1975) as a distinct subregion of the subcommissural ventral basal forebrain complex and a ventral extension of the globus pallidus (GP). Because of its dense innervation by the ventral striatum [nucleus accumbens (NAc) and olfactory tubercle (OT)], the VP was proposed to be a central node in a ventral basal ganglia circuit for the integration of emotional or “limbic” information (Heimer and Wilson, 1975; Walaas and Fonnum, 1979). The subsequent discovery that the VP densely innervates the MD led to the notion of a ventral “limbic” cortico-striatopallidal-thalamic system for emotional-motor regulation (Heimer et al., 1982) and a ventral analog to a series of similar dorsal basal ganglia circuits for sensory-motor integration (Alexander et al., 1986; O’Donnell et al., 1997; Heimer, 2003). Around the same time, VP innervation of the reticular formation and extrapyramidal motor systems was proposed as a key regulator of motivational motor output (Mogenson et al., 1980; Zahm and Brog, 1992; Mogenson et al., 1993; Kalivas and Nakamura, 1999). More recent work strongly suggests that the VP is a central hub for the processing of reward related information and the regulation of motivated states, which are mediated in large part by its connectivity with the dopamine system of the ventral mesencephalon (Haber et al., 1985; Zahm, 1989, 2016; Groenewegen et al., 1993). The VP is also densely interconnected with neighboring basal forebrain regions, hypothalamic nuclei, brainstem areas, and also more sparsely connected with cortical, allocortical, and thalamic regions involved in emotional and motivational regulation (Russchen et al., 1985; Fuller et al., 1987; Vertes, 2004; Vertes and Hoover, 2008; Tripathi et al., 2013). Collectively, these anatomical observations have given rise to the notion that the VP is a central regulator of reward and motivational processing in its own right and not simply a relay and output structure of ventral basal ganglia circuits (Kretschmer, 2000; Root et al., 2015).

The VP shares many similarities with the internal and external segments of the GP such as interconnectivity with the striatum, thalamus, and ventral mesencephalon, and the presence of cortically projecting neurons (Haber et al., 1985; Groenewegen et al., 1993; Zaborszky et al., 2012). However, the VP also projects broadly to regions outside of classically defined basal ganglia circuits in the hypothalamus, extended amygdala, and brain stem (Haber et al., 1985; Tripathi et al., 2013). The VP does not contain a clear segmentation between internal and external segments that give rise to direct and indirect pathways for the differential regulation of movement and motivated states that are prominently seen in the dorsal basal ganglia (Gerfen and Surmeier, 2011; Smith et al., 2013; Kupchik et al., 2015). While anatomical direct and indirect pathway segregation has been proposed to exist at the level of the VP (Sesack and Grace, 2010), recent work clearly demonstrates that such circuits do not originate from ventral striatal dopamine D1 or D2 receptor expressing medium spiny neurons (D1-/D2-MSNs; Kupchik et al., 2015). D1- and D2-MSNs provide densely intermixed projections to the VP (Lu et al., 1998; Smith et al., 2013; Kupchik et al., 2015), and both innervate populations of VP neurons that innervate the ventral mesencephalon (indirect pathway), or the MD (direct pathway; Kupchik et al., 2015; Leung and Balleine, 2015).

**Figure 1:**
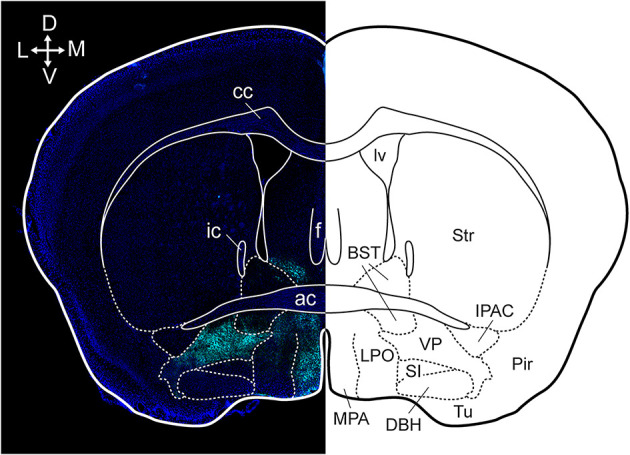
Anatomical localization of the VP in a coronal section. Dense substance P immunoreactivity (cyan) delineates the VP from surrounding basal forebrain regions and the section is counterstained with DAPI (blue). cc, corpus callosum; ic, internal capsule; ac, anterior commissure; f, fornix; lv, lateral ventricle; BST, bed nucleus of the stria terminalis; Str, striatum; MPA, medial preoptic area; LPO, lateral preoptic area; SI, substantia innominate; DBH, horizontal limb of the diagonal band; VP, ventral pallidum; Tu, tubercle; Pir, piriform cortex; IPAC, interstitial nucleus of the posterior limb of the anterior commissure. Arrows indicate the orientation of the brain along the dorsoventral (DV) and mediolateral (ML) axes. Figure adopted from Heinsbroek et al. (2020) and Paxinos and Franklin (2004).

**Figure 2:**
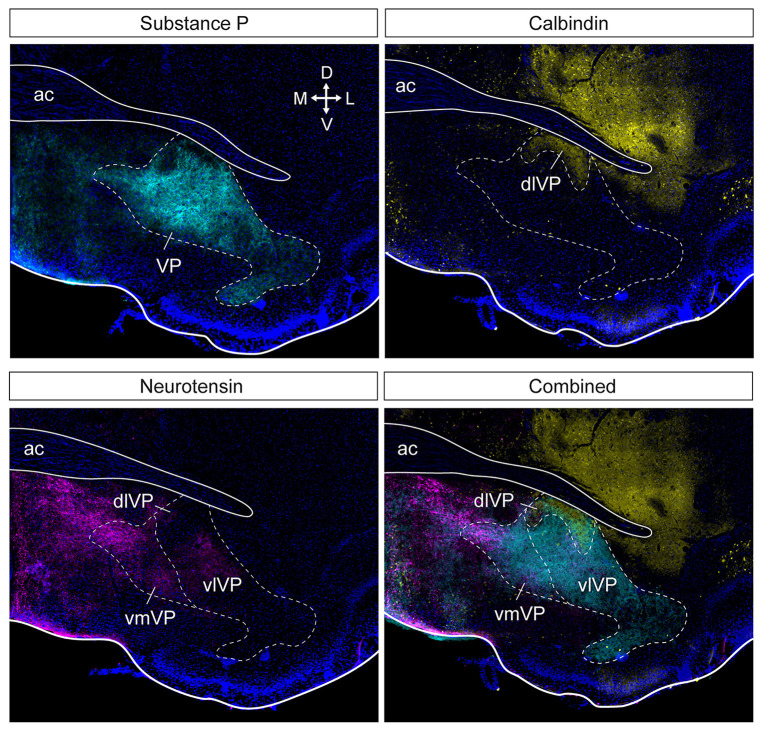
Subdivision of the ventral pallidum into several functionally and histochemically distinct subregions. The series of adjacent sections (at bregma) were stained for substance P, calbindin, and neurotensin. Substance P (cyan) immunostaining shows the borders of the entire VP (top left). Calbindin (yellow) delineates the dlVP and ventral striatum (top right). Dense neurotensin labeling (magenta) outlines the vmVP and more sparse labeling or lack of labeling indicate the vlVP and dlVP (bottom left). The stains combined show the distinct subterritories of the VP. ac, anterior commissure; dlVP, dorsolateral ventral pallidum; vmVP, ventromedial ventral pallidum; vlVP, ventrolateral ventral pallidum. Arrows indicate the orientation of the brain along the dorsoventral (DV) and mediolateral (ML) axes. Sections are counterstained with DAPI (blue). Figure adopted from Zahm et al. (1996) and Heinsbroek et al. (2020).

**Figure 3:**
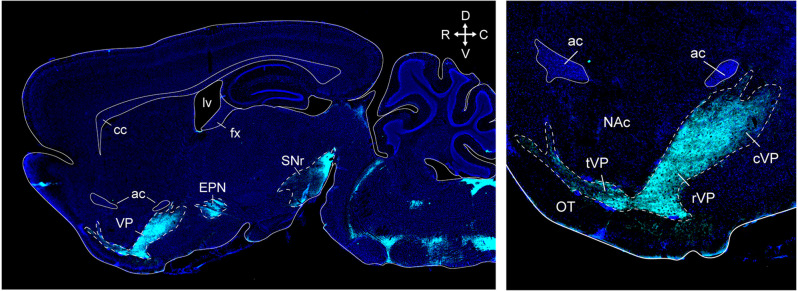
VP and other basal ganglia structures in a sagittal section of the mouse brain. Supstance P immunostaining (cyan) clearly outlines the VP, endopeduncular nucleus, and substantia nigra (top left). Distinct ventral pallidum subregions along the rostrocaudal axis can be seen at higher magnification in this section (top right). The tubercular rostral VP introdes the olfactory tubercle as “fingerlike extensions”. The rostral VP is located ventral to the NAc, and the caudal VP is located posterior to the NAc and ventral to the anterior commissure. ac, anterior commissure; cc, corpus callosum; fx, fornix; lv, lateral ventricle; cVP, caudal ventral pallidum; rVP, rostral ventral pallidum; tVP, tubercular ventral pallidum; OT, olfactory tubercle; NAc, nucleus accumbens; EPN, endopeduncular nucleus; SNr, substantia nigra pars reticulata. Arrows indicate the orientation of the brain along the rostrocaudal (RC) and mediolateral (ML) axes. Sections are counterstained with DAPI (blue). Figure adopted from Zahm et al. (1996) and Heinsbroek et al. (2020).

**Figure 4:**
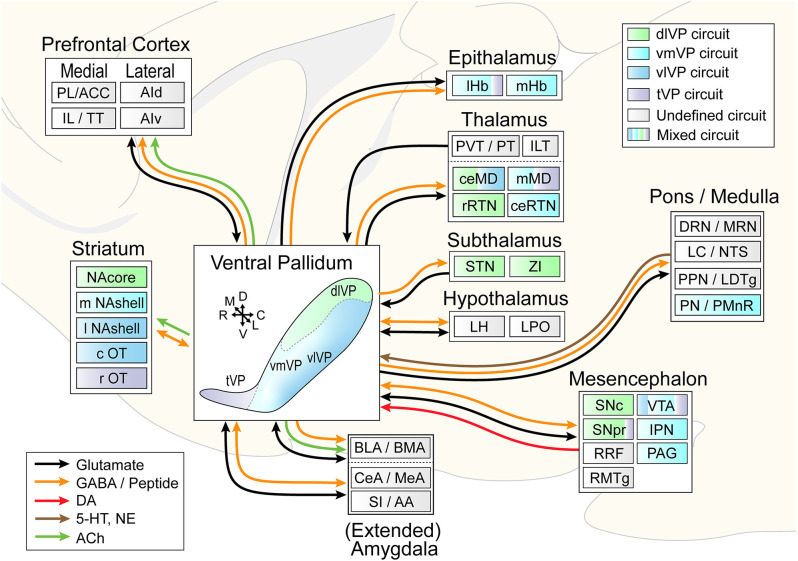
Circuit diagram illustrating VP subcircuit connectivity. In the center, the distinct VP subregions are shown by different colors that are preserved throughout the diagram to indicate known preferential subcircuit connectivity patterns. The ventromedial (light blue) and ventrolateral (dark blue) VP are shown as a color gradient in this representation from medial (back) to lateral (front), but these regions do occupy the entire mid- to caudal extent of the ventral VP. Connected areas are grouped by anatomical region. Arrows indicate VP efferents and afferents, and different colors represent different neurotransmitters. Multicolored regions receive inputs from two or three different VP subregions, and gray regions have not been investigated for subcircuit connectivity. ACC, anterior cingulate cortex; PL, prelimibic cortex; IL, infralimbic cortex; TT, tenia tectum; AId/AIv, dorsal and ventral anterior insular cortex; lHb/mHb, lateral and medial habenula; PVT/PT, paraventricular and paratenial nucleus of the thalamus; ILT, intralaminar nuclei of the thalamus; ceMD/mMD, central and medial dorsomedial nuclei of the thalamus; rRTN/ceRTN, rostral and central parts of the reticular thalamic nucleus; STN, subthalamic nucleus; ZI, zona incerta; BLA/BMA, basolateral and basomedial nucleus of the amygdala; CeA/MeA, central and medial nucleus of the amygdala; SI/AA, substantia innomunate and anterior amygdaloid area; DRN/MRN, dorsal and median raphe nuclei; LC/NTS, locus coeruleus and nucleus of the solitary tract; PPN/LTDg, pedunculopontine nucleus and laterodorsal tegmentum; PN/PMnR, pontine nuclei and paramedian raphe nucleus; SNc/SNr, substantia nigra pars compacta and pars reticulata; RRF, retrorubral field; RMTg, rostromedial tegmentum; VTA, ventral tegmental area; IPN, interpeduncular nucleus; PAG, periaqueductal gray. Arrows indicate the orientation of the brain along the rostrocaudal (RC), dorsoventral (DV), and mediolateral (ML) axes.

Anatomical tracing and immunohistochemical studies have revealed the existence of several VP subregions that partake in distinct transpallidal ventral basal ganglia circuits (Zahm and Heimer, 1990; Groenewegen et al., 1993). The VP is demarcated from surrounding basal forebrain structures by its dense immunoreactivity for the neuropeptides enkephalin and substance P (Zahm, 1989). Additional labeling for the calcium-binding protein calbindin delineates a dorsolateral segment (dlVP) that is preferentially innervated by the nucleus accumbens core (NAcore), and labeling for the neuropeptide neurotensin defines a ventromedial segment (vmVP) that is innervated by the medial nucleus accumbens shell (NAshell; Zahm, 1989; Zahm and Heimer, 1990; Heimer et al., 1991). The dlVP densely projects to the subthalamic nucleus (STN), and substantia nigra, while the vmVP prominently innervates the ventral tegmental area (VTA; Zahm, 1989; Zahm and Heimer, 1990). Other VP subregions without dense calbindin or neurotensin labeling have been proposed and these include a ventrolateral segment (vlVP) that is preferentially innervated by the lateral NAshell and lateral OT (Groenewegen et al., 1993; Zhou et al., 2003), and a rostral “tubercular” extension of the VP (tVP) that consists of “fingerlike extensions” that intrude the rostral OT and preferentially interconnect with this region (Tripathi et al., 2013). Importantly, each of these pallidal subregions has been shown to innervate distinct territories of communal downstream areas and thus likely contributes to separate transpallidal subcircuits (Groenewegen et al., 1993). For instance, the vmVP innervates the medial aspect of the MD which in turn innervates the ventromedial prefrontal cortex, whereas the dlVP innervates central parts of the MD that innervate prelimbic and anterior cingulate regions of the prefrontal cortex (Groenewegen, 1988; O’Donnell et al., 1997). In addition to these subregions, differences between the rostral and caudal VP have been reported. The rostral VP innervates the medial NAcore and NAshell, while the caudal VP innervates the dorsal NAcore (Churchill and Kalivas, 1994), and rostral and caudal VP neurons have distinct membrane properties (Kupchik and Kalivas, 2013). A detailed anatomical delineation of the VP and its subregions is shown in Figures 1–3, and subregion-specific VP connectivity is summarized in Figure 4.

### Cellular heterogeneity

Although the VP is canonically considered a GABAergic relay and output structure of the ventral basal ganglia, it contains substantial populations of glutamatergic (Manns et al., 2001; Hur and Zaborszky, 2005; Geisler et al., 2007) and acetylcholinergic (ACh) projection neurons (Mesulam et al., 1983; Faget et al., 2018; Tooley et al., 2018). VP glutamate and GABA neurons are largely segregated populations (Knowland et al., 2017; Faget et al., 2018; Tooley et al., 2018; Heinsbroek et al., 2020), whereas VP cholinergic neurons co-express markers for glutamate and GABA neurotransmission. The cholinergic neurons of the VP (VP_ACh_) comprise a module of the larger basal forebrain cholinergic system that spans multiple regions and includes the basal nucleus of Meynert (Mesulam et al., 1983; Heimer et al., 1991; Zaborszky et al., 2012, 2015). VP_ACh_ neurons specifically project to the basolateral amygdala (BLA) and prefrontal cortex, and more sparsely to the NAc and medial amygdala (Faget et al., 2018). Compared to neighboring basal forebrain regions, a relatively large subpopulation of VP_ACh_ neurons that project to the BLA co-express the glutamate transporter 3 (Vglut3; Nickerson Poulin et al., 2006), and most VP_ACh_ neurons co-express the GABA synthesizing enzyme GABA decarboxylase 2 and the vesicular GABA transporter (Saunders et al., 2015).

The majority (~74%) of VP neurons are GABAergic (VP_GABA_) and this cell group can be further partitioned into multiple subpopulations (Faget et al., 2018; Heinsbroek et al., 2020). One subpopulation of VP_GABA_ neurons expresses the opioid neuropeptide precursor pro-enkephalin (Penk; VP_Penk_) and projects to the VTA, MD and the striatum (Kalivas et al., 1993; Churchill and Kalivas, 1994; Engeln et al., 2022). In the GP, neurons expressing Penk represent a functionally distinct subpopulation of GABAergic cells (Mallet et al., 2012), that co-express the transcription factor Neuronal Per-Arnt-Sim domain 1 (Npas1) and are preferentially innervated by striatal D1-MSNs (Abdi et al., 2015; Cui et al., 2021a). Whether VP_Penk_ neurons also co-express Npas1 remains to be determined, but VP_Penk_ do receive preferential innervation from NAc D1-MSNs (Heinsbroek et al., 2020). Within the GP complex heterogeneity of GABAergic neurons has been described, but similar detailed phenotyping has yet to be performed for the VP (Saunders et al., 2018). Nevertheless, *in situ* hybridization (Allen Brain Atlas) confirms that the main genes associated with different types of GABAergic pallidal neurons (e.g., Npas1, Penk, PV, Lhx6, FoxP2, Kcng4, Npr3, Npy2r, Sox6, Nkx2.1, and Dbx1) are also expressed in the VP (Lein et al., 2007; Abdi et al., 2015; Abecassis et al., 2020; Cui et al., 2021b). However, there is some evidence for differences between these neurons in the VP and GP. For instance, while GP Npas1 neurons preferentially innervate the striatum and cortex (Hernandez et al., 2015; Abecassis et al., 2020), VP_Npas1_ neurons provide only sparse inputs to the NAc and do not innervate the cortex. Instead, VP_Npas1_ neurons densely innervate the MD, lateral habenula (lHb), and lateral hypothalamus (LH; Morais-Silva et al., 2022). Furthermore, VP_Npas1_ neurons are not an exclusively GABAergic population and express genes for glutamatergic neurotransmission (Morais-Silva et al., 2022). The VP also contains neuropeptide Y, somatostatin, and galanin neurons, which are likely GABAergic (Johansson et al., 1984; Perez et al., 2001; Zaborszky et al., 2012).

Glutamatergic VP neurons can be distinguished based on their expression of different vesicular glutamate transporters. VP glutamate neurons that express Vglut2 (VP_Glu_) comprise about 15%–20% of the total neuronal population and are mostly localized to the vmVP (Hur and Zaborszky, 2005; Geisler et al., 2008; Faget et al., 2018; Heinsbroek et al., 2020). The remaining Vglut3 VP neurons are likely cholinergic. Overall, VP_Glu_ and VP_GABA_ neurons have been found to project to largely overlapping downstream structures with the exception of the NAc, STN, and basomedial amygdala which are exclusively innervated by VP_GABA_ neurons (Faget et al., 2018; Tooley et al., 2018; Wulff et al., 2019). VP_Glu_ and VP_GABA_ neurons also receive monosynaptic inputs from a largely overlapping network of brain regions (Tooley et al., 2018; Heinsbroek et al., 2020; Stephenson-Jones et al., 2020).

A substantial proportion of VP neurons express the calcium-binding protein parvalbumin (PV; VP_PV_) and have high spontaneous activity and fast spiking characteristics (Celio, 1990; Gritti et al., 2003; Knowland et al., 2017; Tooley et al., 2018). Interestingly, subpopulations of both glutamatergic and GABAergic VP neurons express PV (Knowland et al., 2017; Tooley et al., 2018), but these neurons differentially innervate downstream structures. VP_PV_ projections to the lHb are mostly glutamatergic. By contrast, VP_PV_ efferents to the VTA are more evenly composed of glutamatergic and GABAergic axons, but GABAergic VP_PV_ neurons preferentially innervate GABAergic VTA neurons (VTA_GABA_), whereas VTA dopamine neurons (VTA_DA_) are innervated by glutamatergic and GABAergic VP_PV_ axons. Based on their projection target VP_PV_ neurons also receive different inputs. VTA projecting VP_PV_ neurons are preferentially innervated by the basal ganglia and central amygdala, whereas lHb projecting VP_PV_ neurons receive preferential inputs from the bed nucleus of the stria terminalis (Knowland et al., 2017). In addition to PV, VP neurons also express the calcium-binding proteins calretinin, calbindin, and secretagogin (Gritti et al., 2003; Zaborszky et al., 2012), and both calbindin and calretinin are expressed in a substantial number of VP_Glu_ neurons (McKenna et al., 2021).

VP neurons can be distinguished by their differential expression of dopamine D1, D2, and D3 receptors (Napier and Maslowski-Cobuzzi, 1994; Pribiag et al., 2021). Dopamine D3 receptor expressing neurons (VP_Drd3_) form a heterogeneous subpopulation of VP neurons (40%) that mostly comprises VP_GABA_ neurons but includes sparse numbers of VP_Glu_ and VP_ACh_ neurons. VP_Drd3_ neurons that project to the lHb are preferentially innervated by the lateral NAshell and basomedial amygdala, while VTA-projecting VP_Drd3_ neurons receive preferential input from the central amygdala (Pribiag et al., 2021).

## Part 2: Role of the ventral pallidum in motivated behaviors

### Reinforcement learning, reward processing, and salience attribution

The VP is strongly implicated in reinforcement learning. Positive reinforcement describes the process whereby the presentation of a stimulus increases the probability of behavioral response (e.g., obtain a food reward), whereas negative reinforcement describes situations where an aversive stimulus increases the probability of behavioral responding for the removal of that stimulus (e.g., avoid a foot shock; Koob, 2013). Reinforcement learning involves outcome prediction, and the monitoring of *prediction errors*: discrepancies between expected and experienced outcomes, which serve to optimize behavioral strategies towards maximizing future reward (Rescorla and Wagner, 1972; Schultz, 2016). Over successive trials as outcomes become predictive, prediction error coding neurons shift their activity from reward delivery to conditioned reward-predictive stimuli. Rewards that are larger than expected drive a *positive prediction error* that acts to update reward value and reinforce behavior, whereas smaller than expected reward magnitude or reward omissions drive a *negative prediction error* (a reduction or pause in the activity of prediction error coding neurons; Schultz, 2016). Reward prediction error (RPE) coding was first described in VTA_DA_ neurons (Schultz et al., 1997), but has since also been found encoded in distributed neuronal populations across the brain that innervate the VTA. Importantly, only neurons in the VP, LH, and VTA show complete RPE coding (i.e., encode both positive and negative RPE; Tian et al., 2016).

Reward describes the motivational properties of a stimulus that promote an approach towards, engagement with and/or consumption of it (e.g., food; Schultz, 2016). VP neurons were first shown to encode reward in studies by Tindell and colleagues, who reported that a large majority of recorded VP neurons respond to reward predictive conditioned stimuli and that their activity scales with the incentive value of the associated reward (i.e., the anticipated magnitude of pleasurable experience derived from it; Tindell et al., 2004, 2005). For instance, while concentrated salt solutions typically have low incentive value, salt deprivation makes this reward more desirable and consequently increases VP neuron activity to stimuli that predict this reward (Tindell et al., 2006, 2009). Consequently, optogenetic inhibition of the VP reduces salt seeking under these conditions (Chang et al., 2017). Subsequent studies have now firmly established that VP neurons encode the value of reward-associated conditioned stimuli. The magnitude of VP neuron activation to cues predicts the motivation to obtain the associated reward, and more pronounced activation of VP neurons is associated with shorter latencies to reward retrieval and consumption (Tachibana and Hikosaka, 2012; Richard et al., 2016; Fujimoto et al., 2019). These findings are corroborated by the observation that pharmacological or optogenetic inhibition of VP neurons disrupts reward processing and leads to increased task errors and longer latencies to reward consumption. Interestingly, the encoding of incentive value by VP neurons is generally faster and more accurate than that of “upstream” ventral striatal neurons (Ambroggi et al., 2011; Richard et al., 2016; Fujimoto et al., 2019; Ottenheimer et al., 2019), which suggests a more central role of the VP in reward processing than previously thought.

VP neurons with increased and reduced responses to reward-predictive stimuli have been reported (Tachibana and Hikosaka, 2012; Richard et al., 2016). Recent work shows that increased responses can be attributed to a subset of VP_GABA_ neurons, whereas VP_Glu_ neurons largely correspond to the cells that respond with decreased activity (Stephenson-Jones et al., 2020). In addition, opposite responses (i.e., decreases in VP_GABA_ activity and increases in VP_Glu_ activity) are observed in response to aversive stimuli and associated conditioned cues (Stephenson-Jones et al., 2020). Functional optogenetic manipulations show that VP_Glu_ neurons are required to avoid punishment, and that VP_GABA_ neurons drive reward-seeking (Stephenson-Jones et al., 2020). Thus, VP_GABA_ and VP_Glu_ neurons mediate positive and negative reinforcement, respectively. Importantly, the encoding of positive and negative reinforcement by VP neurons has also been shown in non-human primates (Saga et al., 2017). In line with the observation that VP neurons encode RPEs (Tian et al., 2016), subsets of both VP_Glu_ and VP_GABA_ neurons shift their responding from reward delivery to reward-conditioned cues after learning stimulus-reward associations, and show prediction errors to deviations from expected pleasant or aversive outcomes (Stephenson-Jones et al., 2020). A subsequent study has shown that VP neurons also encode RPEs that reflect the delivery of preferred vs. non-preferred rewards, and that this RPE reduces in size when a single reward is given over multiple trials in a row (Ottenheimer et al., 2020b). In addition, RPE coding has been observed in non-human primates, where it was shown to be specifically mediated by a subpopulation of transiently active VP neurons whose activity predicts the learning rate across a task (Kaplan et al., 2020). Interestingly, RPE coding by VP neurons appears to be highly conserved in vertebrates, as it has also been observed in the VP of songbirds (Chang et al., 2019).

Through reinforcement learning, conditioned stimuli become powerful drivers of motivated behavior and attain incentive value. The process whereby rewards, and their associated cues, produce “wanting” or motivation for that reward is often termed “incentive sensitization” and can be measured using Pavlovian autoshaping or “sign tracking” tasks where incentive value is measured by the duration of an approach towards (or interaction with) a reward predictive stimulus. Individual differences in incentive sensitization are associated with differences in DA signaling (Flagel et al., 2011) and are predictive of resistance to extinction and perseverant responding to cues when rewards are omitted (Robinson et al., 2014; Flagel and Robinson, 2017). In addition, animals that show high salience attribution to conditioned stimuli show increased choice for drugs over non-drug rewards (Tunstall and Kearns, 2015). Thus, the process of incentive sensitization may explain why reward associated cues can become potent drivers for addictive behaviors (e.g., continued use despite negative consequences, and cue-induced craving and relapse) (Robinson and Berridge, 1993). VP neurons play a prominent role in incentive value attribution. Animals with high “sign tracking” phenotypes show increased VP population activity during the presentation of conditioned cues, and the degree of VP activation correlates with enhanced levels of engagement with these cues (Ahrens et al., 2016). Regional differences have been observed in the activity of VP neurons with sustained activity throughout cue presentations. While rostral VP neurons show a predominant sustained inhibitory response to cues, caudal VP neurons show a more heterogeneous response characterized by both sustained inhibition and excitation (Ahrens et al., 2018). These findings indicate substantial contributions of the VP to incentive sensitization and are corroborated by the finding that the chemogenetic inhibition of VP neurons interferes with incentive value attribution and the development of “sign tracking” behavior (Chang et al., 2015). Incentive salience can also be measured using Pavlovian-instrumental transfer models wherein the presentation of a Pavlovian conditioned stimulus that predicts reward delivery produces an increase in responding for that same reward in an operant task and thus permits measurement of the incentive value of the conditioned stimulus (Cartoni et al., 2016). The VP and its afferents from the NAshell and efferents to the MD and VTA are critically important for the processing of incentive value in this model (Leung and Balleine, 2013, 2015). Finally, incentive value attribution to cues can also be measured using conditioned reinforcement tasks wherein a reponse-contingent conditioned stimulus is presented but its associated reward is omitted. Conditioned reinforcement is widely used in cue-induced reinstatement tests (e.g., tests that measure relapse to drug-seeking behavior in SUD models) and is critically dependent on neuronal activity in the VP (Root et al., 2015). The role of the VP in drug relapse models is discussed in more detail below.

VP neurons track reward value under choice conditions, where their activity predicts choices for preferred over non-preferred rewards. Under these conditions, VP neuronal activity is modulated by the satiety state of an animal, and optogenetic stimulation of VP neurons is capable of shifting choice towards the non-preferred reward (Ottenheimer et al., 2020a). Thus, VP neurons process reward-specific information to inform choices. In line with this observation, VP_GABA_ neurons regulate decision-making under risky conditions where the delivery of preferred large rewards is intermittently punished with a foot shock. Chemogenetic inhibition of VP_GABA_ neurons results in a shifted preference towards otherwise less desired low-risk and low reward choices (Farrell et al., 2021).

VP neurons also regulate the rewards derived from social behaviors and are activated during social interaction and exposure to social novelty (Kim et al., 2015; Gomez-Gomez et al., 2019). Indeed, vasopressin 1 receptors have long been known to regulate the formation of long-term partner preference in the VP of monogamous rodents (Pitkow et al., 2001; Lim and Young, 2004; Lim et al., 2004). However, vasopressin projections from the extended amygdala to the VP also regulate social reward in non-monogamous rats (DiBenedictis et al., 2020). The VP similarly plays a crucial role in the rewarding effects of social play. Pharmacological inhibition of VP activity reduces social interaction and social play in rats, and vasopressin produces sex-specific changes in play behavior (Khan et al., 2020; Lee et al., 2021). Social reward processing in the VP is disrupted by exposure to severe stress (e.g., in depression models), which is discussed in section “Part 6: Experience dependent changes in ventral pallidal circuit function.”

Combined, these findings strongly implicate VP activity in reward processing, and this is supported by pharmacological disinhibition of the VP or optogenetic stimulation of VP_GABA_ neurons which produces a rewarding state and leads to the formation of a conditioned place preference (CPP; Faget et al., 2018; Reichard et al., 2019b). In addition, optogenetic stimulation of the VP or VP_GABA_ neurons is acutely reinforcing and produces a real-time place preference (RTPP) when animals are allowed to choose between a stimulated or control chamber (Zhu et al., 2017; Faget et al., 2018; Tooley et al., 2018; Stephenson-Jones et al., 2020; Yao et al., 2021). Operant responding for electrical intracranial self-stimulation (ICSS) of the VP is also highly reinforcing, and this is recapitulated by optogenetic ICSS of VP_GABA_, but not VP_PV_ or VP_Glu_ neurons (Panagis and Spyraki, 1996; Knowland et al., 2017; Faget et al., 2018).

### Hedonic processing and food consumption

Whereas DA regulates the incentive salience and motivation or “wanting” of rewards, hedonic pleasurable experiences or “liking” of rewards is mediated by endogenous opioids and other neuropeptides (Berridge and Kringelbach, 2015). “Wanting” is measured by the motivation to obtain or consume a reward (e.g., by measuring operant responses or food intake), while hedonic responses are measured from innate orofacial movements that reflect “liking” (e.g., tongue protrusions in response to sweet taste) or “disliking” (e.g., mouth gaping responses to bitter tastes) that are highly conserved across mammalian species (Berridge and Kringelbach, 2015).

The differential regulation of food “wanting” and “liking” has been elegantly shown in the VP (Olney et al., 2018). Increases in “wanting” independent of the caloric value of food or hunger state of an animal are observed following pharmacological disinhibition of the VP with GABA_A_ antagonists (Stratford et al., 1999; Smith and Berridge, 2005; Reichard et al., 2019a), and microinjections of mu opioid receptor (MOR) agonists or delta opioid receptor (DOR) antagonists in the VP (Smith and Berridge, 2005; Shimura et al., 2006; Inui and Shimura, 2014). The resulting disinhibition of the VP produces compulsive food “wanting” and gnawing behavior that is blocked by the lesioning of the LH (Stratford and Wirtshafter, 2012) and by dopamine D2 receptor antagonists (Reichard et al., 2019a). Hunting, consummatory, and gnawing behaviors are also seen during optogenetic stimulation of VP_GABA_ neurons, and their projections to the midbrain periaqueductal gray (Zhu et al., 2017; Roman-Ortiz et al., 2021). In addition, the inhibition of VP_GABA_ neurons or stimulation of VP_Glu_ neurons reduces sucrose consumption (Yao et al., 2021).

In contrast to food “wanting”, hedonic food “liking” is regulated by the VP in a subregion-specific manner. MOR or orexin (ORX) receptor activation in the caudal VP strongly amplifies hedonic reactions to sweet tastes (Smith and Berridge, 2005; Ho and Berridge, 2013). This VP region is reciprocally connected to a related hedonic subregion in the NAshell and activity in both nodes of this hedonic network is required for the pleasurable experience of reward (Smith and Berridge, 2007; Vachez et al., 2021). By contrast, MOR activation in the rostral VP reduces hedonic “liking” (Smith and Berridge, 2005). Interestingly, of all the regions identified to date that regulate hedonic processing, the caudal VP is impacted the most by irreversible lesions and pharmacological manipulations, which transform pleasurable responses to sweet rewards into disgust (Cromwell and Berridge, 1993; Shimura et al., 2006; Ho and Berridge, 2014). Thus, caudal VP neurons are not only required for the amplification of hedonic states by neuropeptide systems, but also critically involved in the regulation of normal hedonic processing. Collectively, these findings show that the VP is a central node in a neural network for the experience of pleasure.

### Drug reward, craving, and relapse to drug-seeking

SUDs are characterized by a pervasive motivation to seek and take drugs despite negative consequences and a loss of interest in natural rewards. Drugs of abuse usurp the neural circuits that regulate reward processing, reinforcement learning, and hedonic processing, and produce persistent changes in the functioning of the VP. SUDs are studied using a variety of animal models (Kuhn et al., 2019; Venniro et al., 2020; Heinsbroek et al., 2021). Non-contingent (experimenter administered drug) models include behavioral sensitization for examining potentiated behavioral responses to repeated injections of drugs of abuse and CPP which measures the formation and expression of Pavlovian context-drug reward associations. Response-contingent (self-administration) models assess volitional consumption and motivation to seek drugs using operant (instrumental) tasks. The role of the VP in SUDs has been summarized in detail elsewhere (Root et al., 2015; Kupchik and Prasad, 2021), but a brief summary of the literature and discussion of recent findings is provided below.

The VP is critically important for the development of behavioral sensitization to opioids and psychostimulants (Johnson et al., 2000; Chen et al., 2001; Dallimore et al., 2006; Mickiewicz et al., 2009; Creed et al., 2016). Behavioral sensitization is linked to changes in VP functioning, and its expression can be reduced by normalizing GABA transmission or blocking glutamate or opioid neurotransmission in the VP (Chen et al., 2001; McDaid et al., 2005, 2006; Mickiewicz et al., 2009; Creed et al., 2016). The circuit mechanisms by which the VP regulates behavioral sensitization remain largely unclear, but likely involve interactions with the DA system and changes in VP GABA- and glutamatergic neurotransmission (Chen et al., 2001; McDaid et al., 2005; Creed et al., 2016; Stout et al., 2016). Recent work also suggests an involvement of dlVP projections to the STN in conditioned locomotor responses to an amphetamine-paired chamber (Nakata et al., 2022). In addition to behavioral sensitization, the VP is critically involved in the conditioned rewarding effects of opioids, psychostimulants, and alcohol in CPP and self-administration models (Hubner and Koob, 1990; Hiroi and White, 1993; June et al., 2003; Skoubis and Maidment, 2003; Dallimore et al., 2006). Furthermore, local VP infusions of psychostimulants are rewarding and produce a CPP, and morphine microinjections in the VP augment a subsequent CPP to systemic morphine injections (Gong et al., 1997; Zarrindast et al., 2007).

The VP has been studied extensively for its regulation of drug craving and relapse to drug-seeking in self-administration models. Relapse is studied under drug-free conditions after forced abstinence in the home cage, or a period of extinction training, and can be reliably evoked with drug-conditioned cues, drug-associated contexts, exposure to stressful stimuli, or small priming doses of drugs (Kuhn et al., 2019; Heinsbroek et al., 2021). Relapse requires neuronal activity in a “final common pathway” that includes the prelimbic prefrontal cortex, NAcore, and dlVP (McFarland and Kalivas, 2001; McFarland et al., 2003; Kalivas, 2009; Stefanik et al., 2013a,b). Interestingly, relapse to drug cues is mediated by the rostral VP, whereas the caudal VP regulates relapse produced by priming injections of cocaine (Mahler et al., 2014b).

Relapse to cocaine-seeking is differentially regulated by VP neuronal subpopulations. Calcium imaging shows that VP_GABA_ neurons respond with increased tonic activity during cue-induced reinstatement (an elevation in the frequency of calcium events across a session), whereas VP_Penk_ neurons respond with an increase in phasic activity around nose pokes for cocaine during reinstatement (Heinsbroek et al., 2020). These findings indicate that VP_GABA_ and VP_Penk_ neurons may differentially regulate relapse to cocaine seeking. Indeed, while chemogenetic activation of VP_GABA_ or VP_Penk_ neurons increases cocaine seeking in extinguished mice in the absence of drug-conditioned cues, stimulation of VP_Penk_ but not VP_GABA_ neurons potentiates cue-induced reinstatement. Thus, VP_Penk_ neurons may be a more potent regulator of cocaine relapse than the overall GABAergic VP population. An intriguing possibility is that enkephalin released by VP_Penk_ neurons contributes to an increase in VP enkephalin transmission that is known to promote relapse to cocaine-seeking (Tang et al., 2005; Kupchik et al., 2014; Creed et al., 2016; Heinsbroek et al., 2017). In contrast to VP_GABA_ and VP_Penk_ neurons, VP_Glu_ neurons show increased tonic activity after extinction training, which suggests these cells may negatively regulate the motivation to seek cocaine. Indeed, chemogenetic activation of VP_Glu_ neurons reduces responding under both extinction and reinstatement conditions (Heinsbroek et al., 2020). Calcium imaging also shows increased tonic activity in VP_Drd3_ neurons during cocaine relapse after a period of forced abstinence (Pribiag et al., 2021).

Context-driven reinstatement of alcohol seeking measured using two distinct chambers (ABA model) is mediated by enkephalin signaling in the VP (Perry and McNally, 2013). In this model, chemogenetic VP inhibition reduces, and stimulation of the VP promotes relapse to alcohol seeking and the re-acquisition of alcohol self-administration (Prasad and McNally, 2016). These effects are mediated by overlapping neuronal VP populations. Inhibition of VP_GABA_ neurons reduces relapse but does not affect re-acquisition, whereas inhibition of the mixed population of GABAergic and glutamatergic VP_PV_ neurons reduces both alcohol relapse and re-acquisition (Prasad et al., 2020). In congruence with these findings, a recent study shows that chemogenetic stimulation of VP_GABA_ neurons increases relapse to seeking of the opioid drug remifentanil in this model (Farrell et al., 2022).

Voluntary abstinence from cocaine use following the introduction of negative consequences (i.e., foot shocks) in a different context is also regulated by VP neurons. Under these conditions, chemogenetic inhibition of the VP reduces conflict behavior in rats measured as a hesitancy to lever press or “lever abortions” following the introduction of punishment. In addition, inhibiting the VP reduces drug-seeking when rats are returned to their initial safe training context where they first learned to self-administer cocaine (Farrell et al., 2019). Furthermore, chemogenetic inhibition of VP_GABA_ neurons reduces cue-induced seeking of the opioid remifentanil upon returning rats to the safe context, whereas stimulation of VP_GABA_ neurons increases drug-seeking in both the safe and the punished context (Farrell et al., 2022).

VP neurons show heterogeneous activity during cocaine and alcohol seeking (Root et al., 2010; Ottenheimer et al., 2019), that differs based on their anatomical location (Root et al., 2012, 2013). Overall, neurons in the dlVP are more active than those in the vmVP when rats approach an operandum to self-administer cocaine (Root et al., 2012). In addition, compared to the vmVP, the activity of dlVP neurons is more homogeneous between an approach and operant response for cocaine (Root et al., 2013). These findings may indicate that the dlVP is a more prominent driver of cocaine seeking, but this requires further examination. Drug exposure also disrupts VP reward coding. VP neurons respond reliably to conditioned stimuli that predict sucrose reward in either instrumental or Pavlovian tasks (Richard et al., 2018), but fail to show responding to instrumental stimuli that predict alcohol reward. Furthermore, alcohol pre-exposure disrupts VP reward processing for sucrose, and results in augmented responses to instrumental stimuli and diminished responses to Pavlovian sucrose-conditioned stimuli. This may be explained by reward-seeking becoming more habitual and less dependent on outcomes after exposure to alcohol (Ottenheimer et al., 2019). In addition, VP reward processing is disrupted by repeated systemic administrations of amphetamine, or a single intra-accumbens administration of this drug. Following these treatments, VP neurons shift their responding away from distal reward-predictive stimuli towards more salient temporally proximal cues (Tindell et al., 2006; Smith et al., 2011).

### Negative emotional states and aversion

The VP regulates negative affective states and aversion (for comprehensive reviews, see: Stephenson-Jones, 2019; Wulff et al., 2019). Inhibiting the VP disrupts appropriate behavioral responses to aversive stimuli (Saga et al., 2017), and a real-time place aversion is observed following the optogenetic stimulation of VP_Glu_ neurons and their projections to the VTA and lHb (Faget et al., 2018; Tooley et al., 2018; Liu et al., 2020; Stephenson-Jones et al., 2020; McKenna et al., 2021; Yao et al., 2021). VP_Glu_ has also been shown to increase their firing in response to aversive stimuli, drive adaptive responses to avoid punishment and to attenuate reward-seeking behaviors (Stephenson-Jones et al., 2020). In addition, manipulations that reduce the activity of VP_GABA_ are acutely aversive, but whether inhibition of VP_Glu_ is intrinsically rewarding remains unclear (Faget et al., 2018; Yao et al., 2021). The regulation of aversion by changes in VP_GABA_ and VP_Glu_ activity is likely mediated by a downstream increase in glutamate over GABA neurotransmission in the lHb, VTA, and rostromedial tegmentum (RMTg; Zahm, 2016; Faget et al., 2018; Tooley et al., 2018; Gordon-Fennell and Stuber, 2021). In addition, changes in VP opioid and GABA signaling contribute to negative affect and reduced motivation (Smith and Berridge, 2005; Skirzewski et al., 2011; Creed et al., 2016). However, while VP_Glu_ neurons are generally associated with aversion and learning about unpleasant stimuli, their role extends beyond aversion processing and negative reinforcement. VP_Glu_ neuron activity is increased in response to salient novel stimuli regardless of their valence, and inhibiting these cells not only disrupts threat avoidance but also diminishes interaction with novel objects and unfamiliar mice (Wang et al., 2020). Thus, in addition to regulating aversion, VP_Glu_ neurons regulate salience processing. This may in part be mediated by the arousal promoting function of VP_Glu_ neurons (McKenna et al., 2021).

In line with its role in aversion processing, the VP has been implicated in animal models of anxiety- and depression-like behaviors (Hasenohrl et al., 2000; Chang and Grace, 2014). Pharmacological disinhibition of the VP reduces anxiety measured by time spent in the center of an open field (OF) and time spent in the open arms of an elevated plus maze (EPM), and these effects depend on dopamine D1- and D2-receptor signaling (Reichard et al., 2019b). However, optogenetic or chemogenetic stimulation of VP_GABA_ produces mixed results in anxiety tests with either no effects or increased anxiety in the EPM (Zhu et al., 2017; Li et al., 2021; Roman-Ortiz et al., 2021), or no effects or reduced anxiety in OF and light-dark box tests (Zhu et al., 2017; Li et al., 2021). By contrast, chemogenetic stimulation of VP_Npas1_ neurons augments anxiety in the EPM (Morais-Silva et al., 2022). Given that VP_Npas1_ neurons are capable of releasing glutamate, these effects may in part be mediated by glutamate released in downstream target structures. In line with a role for the VP in anxiety, threatening stimuli produce a sustained inhibition of VP neurons (Moaddab et al., 2021). Furthermore, some data implicate the VP in the processing of conditioned aversive behaviors. Stimulating VP_Penk_ neurons disrupts the acquisition of memory for aversive stimuli (Macpherson et al., 2019), whereas stimulating VP_GABA_ neurons disrupts the expression, but not the acquisition of conditioned fear responses (Roman-Ortiz et al., 2021). However, compulsive gnawing behavior produced by VP_GABA_ stimulation may have interfered with fear expression in this test (Roman-Ortiz et al., 2021).

The VP is activated during social defeat stress, a widely used procedure for modeling a stress-induced depressive-like state (Lkhagvasuren et al., 2014). Increased activity of VP_PV_ neurons is associated with negative affect and motivational deficits following chronic stress, and inhibiting VP_PV_ improves measures of behavioral despair (struggling in a tail-suspension test), as well as post-stress deficits in social reward processing (Knowland et al., 2017). VP_Npas1_ neurons also regulate stress susceptibility, and stimulating these cells increases social avoidance following social defeat stress, while inhibition of these neurons confers protection against the negative effects of social defeat (Morais-Silva et al., 2022).

### Regulation of movement

Following the initial description of the NAc and VP as a limbic-motor interface for the “conversion of motivation into action” by Mogenson et al. (1980, 1993); a large body of research has firmly established a role for the VP in the regulation of exploratory locomotion, and movements related to reward-seeking and consummatory behaviors (for detailed reviews, see: Smith et al., 2009; Root et al., 2015). Recent cell- and circuit-specific manipulations show complex and conflicting results on the regulation of movement by the VP. For instance, increased exploratory movement is seen during the optogenetic stimulation of VP_Glu_ or inhibition of VP_GABA_ neurons (Yao et al., 2021) but chemogenetic stimulation of VP_GABA_ neurons also increases movement (Li et al., 2021). By contrast, other manipulations targeting VP circuits do not elicit changes in locomotor behavior (Stefanik et al., 2013a; Prasad and McNally, 2016; Prasad et al., 2020; Vachez et al., 2021). Thus, more research is necessary to clarify the cell- and circuit-specific VP contributions to the regulation of movement. Of note, VP manipulations produce the most pronounced effects on movement in non-habituated OF arenas or under anxiogenic conditions such as exposure to bright lights (Hooks and Kalivas, 1995; Reichard et al., 2019b), and differences in experimental details such as these likely contribute to conflicting findings in the field.

The VP and its projections to the reticular formation and extrapyramidal motor regions of the brain stem have long been hypothesized to regulate movement (Mogenson et al., 1993), and locomotion produced by the pharmacological disinhibition of the VP can be blocked by the inhibition of the mesencephalic extrapyramidal motor region (the pedunculopontine nucleus and surrounding areas; PPN; Churchill and Kalivas, 1999). However, while PPN retrograde tracer injections produce labeling in the VP (Groenewegen et al., 1993), anterograde tracing studies reveal relatively sparse VP axon labeling in the PPN (Swanson et al., 1984; Haber et al., 1985; Mogenson et al., 1993; Churchill and Kalivas, 1999; Tripathi et al., 2013; Faget et al., 2018). Thus, the VP most likely conveys motivational information to brain stem motor centers through intermediary structures (e.g., the substantia nigra and other higher-order brain stem regions). Indeed, recent work shows that compulsive feeding and gnawing behaviors produced by VP_GABA_ activation depend on the ventrolateral periaqueductal gray (Zhu et al., 2017; Roman-Ortiz et al., 2021). Thus, the VP likely indirectly relays motivational information to the brain stem for the invigoration of movement. In support of this many VP neurons respond strongly to reward-associated stimuli but very few VP neurons are specifically activated during somatic or orofacial movements (Tindell et al., 2004; Ahrens et al., 2016).

VP projections to the MD and VTA are also implicated in the regulation of exploratory movement (Mogenson et al., 1993; Kalivas and Nakamura, 1999). The ventral cortico-striatopallidal-thalamic loop that contains the VP runs parallel to analogous dorsal basal ganglia loops for sensory-motor integration (Alexander et al., 1986; Foster et al., 2021). In these parallel circuits information flows from ventral associative loops to dorsal sensorimotor circuits (Zahm and Brog, 1992), and this provides a mechanism whereby motivational information from the VP can influence the pyramidal motor systems of the cortex and associated basal ganglia loops (Zahm and Brog, 1992; Kalivas and Nakamura, 1999). In addition, similar “upwards” spirals have been described for connections between the striatum and the ventral mesencephalon (Nauta et al., 1978; Haber et al., 2000), and given its interconnectivity with these regions the VP is centrally positioned to influence action selection and the regulation of movement by this system (Heimer, 2003).

## Part 3: Role of ventral pallidal afferents and neurotransmitter systems in motivated behaviors

### GABAergic neurotransmission

GABA is by far the predominant neurotransmitter in the VP, and GABAergic synapses outnumber other synapses five-fold (Chang et al., 1995). GABAergic inputs to VP mostly originate from the NAc, but the VP also receives sparse GABAergic inputs from the VTA, the hypothalamus, and extended amygdala (Walaas and Fonnum, 1979; Zahm and Brog, 1992; Jennings et al., 2013; Taylor et al., 2014; Zhou et al., 2022). Given the density of GABAergic synapses, it is no surprise that pharmacological agents targeting GABA receptors produce profound behavioral responses. Ionotropic or metabotropic GABA antagonists microinjected into the VP increase motivation and produce exploratory locomotor and feeding behaviors, while agonists reduce locomotor activity, feeding, and motivated behaviors (Austin and Kalivas, 1990; Smith and Berridge, 2005; Zahm et al., 2013, 2014).

Drugs of abuse reduce GABA in the VP (Bourdelais and Kalivas, 1990; Tang et al., 2005; Li et al., 2009), and VP GABA is also reduced during relapse to cocaine-seeking (Tang et al., 2005). Conversely, increasing GABA in the VP by blocking transporters or degrading enzymes disrupts heroin reinforcement (Xi and Stein, 2002), and activating VP GABA receptors reduces relapse to cocaine-seeking behavior (McFarland and Kalivas, 2001; McFarland et al., 2003). GABA agonists microinjected into the VP also acutely impair the motivation to seek non-drug rewards (Tachibana and Hikosaka, 2012; Richard et al., 2016). Although the circuit mechanisms whereby a reduction in VP GABA promotes motivation are not entirely clear, the activation of D2 receptors on NAc D2-MSNs may provide an important mechanism of action. Overexpression of this receptor in D2-MSNs reduces GABA transmission in the VP and increases motivation (Gallo et al., 2018).

In addition to synaptic release, GABA in the VP is regulated by astroglia and the proximity of perisynaptic astroglial processes and the GABA transporter GAT-3 to striatopallidal synapses (Kruyer et al., 2022). Surprisingly, the knockdown of astroglial GAT-3 in the VP promotes heroin seeking after extinction training by disrupting astroglial clearance of GABA from D1-VP projections after extinction (Kruyer et al., 2022). Given that D1-VP projections drive drug-seeking, impaired GABA clearance in D1-VP synapses may exacerbate drug-seeking behaviors (Pardo-Garcia et al., 2019).

Recent work has revealed a complex regulation of motivated behavior and drug-seeking by GABAergic D1- and D2-VP inputs to the VP. These findings are discussed in section “Part 4: Role of ventral striatopallidal afferents in the regulation of motivated behaviors”. GABAergic inputs from the VTA to the VP also regulate motivated behavior. Activity in this pathway scales with the magnitude of delivered rewards, and chemogenetic activation of this pathway increases the motivation to obtain rewards in progressive ratio and cued reward-seeking tests (Zhou et al., 2022).

### Glutamatergic neurotransmission

Despite the relatively low abundance of glutamatergic synapses in the VP, this neurotransmitter is critically important for reward processing. VP neurons receive dense glutamatergic input from the STN (Turner et al., 2001), and sparse glutamatergic inputs from prefrontal (prelimbic, infralimbic insular), allocortical (amygdala) and midline thalamic (paraventricular and paratenial) regions that innervate the NAc (with the exception of the hippocampus; Kelley et al., 1982; Fuller et al., 1987; Vertes, 2004; Vertes and Hoover, 2008; Perry and McNally, 2013). The VP is also innervated by VTA Vglut2 neurons (Taylor et al., 2014; Yoo et al., 2016). In addition to these sources of glutamate, VP neurons receive intrinsic synaptic glutamate inputs from local VP_Glu_ neurons (Levi et al., 2020; Stephenson-Jones et al., 2020; McKenna et al., 2021). Furthermore, glutamatergic tone in the VP is regulated by glial glutamate transporters (Wydra et al., 2013; Yang et al., 2022).

Ionotropic glutamatergic neurotransmission in the VP regulates exploratory locomotor behavior and is necessary for cued reward-seeking (Churchill and Kalivas, 1999; Richard et al., 2018), the formation of context-drug reward associations (Dallimore et al., 2006), and the development of sensitized locomotor responses to repeated administration of drugs of abuse (Dallimore et al., 2006). In line with these findings, increasing VP glutamate by blocking astroglial glutamate uptake potentiates relapse to heroin seeking (Yang et al., 2022). However, VP glutamate is also capable of reducing cocaine self-administration and cocaine-primed reinstatement through the activation of type III metabotropic receptors in the VP (Li et al., 2009, 2010). Interestingly, activation of these receptors prevents a cocaine-induced reduction in VP GABA transmission (Tang et al., 2005; Li et al., 2010). Thus, glutamatergic signaling in the VP is capable of nuanced regulation of motivated behaviors and drug-seeking by acting through different receptor systems.

Although the precise role of extrinsic synaptic glutamate release in the VP from limbic circuits remains largely unexplored, there are some indications that corticopallidal and thalamopallial glutamatergic inputs to VP are important for regulating motivated behaviors. For instance, context-induced relapse to alcohol-seeking activates BLA and paraventricular thalamus neurons that project to the VP (Perry and McNally, 2013). Glutamatergic inputs to the VP also play a role in the regulation of aversive states. Blocking glutamate signaling in the VP prevents a reduction in the firing of VTA_DA_ neurons following BLA stimulation after social defeat stress, which implicates a BLA-VP-VTA circuit in the reduced activity of the DA system following a traumatic life experience (Chang and Grace, 2014). Interestingly, this same circuit is also activated during withdrawal from cocaine self-administration, suggesting a shared mechanism through which stress and drug withdrawal produce negative affect and reduce VTA_DA_ activity (Salin et al., 2022). In addition, glutamatergic inputs from the VTA to the VP regulate both reinforcement and negative affect, as optogenetic stimulation of this pathway produces ICSS, as well as aversion in a RTPP test (Yoo et al., 2016).

### Neuromodulation in the ventral pallidum

The VP receives neuromodulatory inputs from a variety of brain regions. These include the monoaminergic nuclei of the brain stem and hypothalamus which provide DA, noradrenalin, serotonin, and histamine, as well as peptidergic inputs from a wide range of brain regions. While the VTA provides relatively sparse DA inputs to the VP (Klitenick et al., 1992; Mengual and Pickel, 2004; Stout et al., 2016; Matsui and Alvarez, 2018), DA provides powerful modulatory action on VP neurons. Local application of DA attenuates responses of most VP neurons to glutamate and GABA (Johnson and Napier, 1997), and DA or D1 receptor agonists injected into the VP increase locomotor activity (Klitenick et al., 1992; Gong et al., 1999). By contrast, D2 receptor agonists reduce locomotor activity (Gong et al., 1999). DA levels in the VP are elevated during cocaine self-administration (Sizemore et al., 2000; Wydra et al., 2013), and compared to the striatum, VP DA clearance is much slower, particularly in the caudal portion of the VP. These data suggest that prolonged DA neuromodulation in the VP plays an important role in the regulation of motivated behaviors (Stout et al., 2016; Pribiag et al., 2021).

The VP receives dense serotonergic innervation from the dorsal raphe (Matsui and Alvarez, 2018), but the regulation of motivated behavior by VP serotonin (5HT) remains largely unclear. 5HT has been shown to hyperpolarize cholinergic VP neurons, and depolarize non-cholinergic neurons (Bengtson et al., 2004), and 5HT levels in the VP rise during cocaine self-administration (Sizemore et al., 2000), which suggests that 5HT may contribute to motivated behavior through the activation of non-cholinergic VP neurons. However, 5HT regulation of VP activity is highly complex due to the expression of many different serotonergic receptors in this region. For instance, the activation of 5HT_2C_ receptors in the VP reduces locomotor activity, possibly through the local inhibition of DA release (Graves et al., 2013). By contrast, presynaptic 5HT_1B_ receptors have been shown to alter the information flow from the NAc to the VP by selectively dampening information from D2-MSNs (Matsui and Alvarez, 2018). This finding may be particularly relevant given the role of D2-MSNs and their projections to the VP in depressive-like behaviors (Francis et al., 2015) and the observation that the rapid antidepressant effects of ketamine are associated with an upregulation of 5HT_1B_ receptors in the VP (Yamanaka et al., 2014).

Multiple neuropeptide transmitter systems converge in the VP. Of these, opioid neuropeptides have been studied most extensively. Opioid neuropeptides bind MOR, DOR, kappa opioid receptors (KOR), and nociceptin receptors, all of which are expressed in the VP (Neal et al., 1999; Le Merrer et al., 2009). NAc D1-MSNs release dynorphin and D2-MSNs release enkephalin into the VP (Lu et al., 1998), and both neuropeptides modulate VP neuronal activity and motivated states. Dynorphin selectively activates KOR and reduces the activity of VP neurons (Mitrovic and Napier, 1995). Interestingly, dynorphin reduces inhibitory inputs onto VP_GABA_ but potentiates inhibitory inputs onto VP_Glu_ neurons (Inbar et al., 2020). Although the role of KOR signaling in the VP has not been widely studied, KOR signaling does not appear necessary for alcohol self-administration (Kemppainen et al., 2012).

Enkephalin acts on both DOR and MOR to reduce the activity of VP neurons (Mitrovic and Napier, 1995). Both MOR and DOR have been found to be expressed pre- and post-synaptically in the VP (Hjelmstad et al., 2013; Kupchik et al., 2014; Creed et al., 2016; Heinsbroek et al., 2017, but see: Olive et al., 1997). VP MOR activation increases exploratory locomotor behaviors, and the VP MOR is necessary for the rewarding and behavioral sensitizing effects of cocaine, morphine, and alcohol (Austin and Kalivas, 1990; Skoubis and Maidment, 2003; Mickiewicz et al., 2009; Kemppainen et al., 2012). In addition, VP MOR activation is also required for relapse to alcohol and cocaine seeking (Tang et al., 2005; Perry and McNally, 2013).

Opioid receptors and enkephalin signaling produce subregion-specific effects in the VP. In the caudal VP MOR activation amplifies the positive hedonic impact or “liking” of sucrose reward while rostral VP MOR activation produces the opposite effect (Smith and Berridge, 2005). MOR and DOR activation similarly increases motivated behaviors in a subregion-specific manner. Rostral MOR activation reduces motivation, while caudal MOR activation profoundly increases motivation as measured by a reduction in the electrical ICSS threshold for responding (Johnson et al., 1993). Similarly, DOR activation produces a greater increase in motivation in the caudal compared to the rostral VP (Johnson and Stellar, 1994). DOR or MOR activation also produces more pronounced consummatory behaviors in the caudal compared to the rostral VP (Smith and Berridge, 2005; Inui and Shimura, 2014).

In addition to opioid peptides, the VP receives dense tachykinin (substance P) and neurotensin inputs from the NAc. Microinfusion of substance P in the VP increases the activity of VP neurons and produces exploratory locomotor behavior, reduces anxiety, promotes cognition, and elicits a rewarding state that produces CPP (Napier et al., 1995; Hasenohrl et al., 2000). Conversely, inhibiting neurokinin 1 receptors in the VP reduces the intrinsic excitability of VP neurons, and produces an aversive state (He et al., 2020). However, blocking Substance P signaling in the VP does not affect VP reward processing (Richard et al., 2018). Neurotensin levels are increased in the VP in chronic cocaine users (Frankel et al., 2008), and local administration of neurotensin increases cocaine-primed reinstatement but attenuates cue-induced reinstatement of cocaine-seeking (Torregrossa and Kalivas, 2008). In drug-naïve animals neurotensin microinjected into the VP is anxiolytic and produces CPP (Ollmann et al., 2015a,b). Based on these findings, VP neurotensin may reduce cue-induced reinstatement by counteracting the aversive and anxiogenic effects produced by drug cues during relapse (Morales-Rivera et al., 2014), but amplify the rewarding and relapse promoting effects of cocaine.

The VP receives prominent ORX input from the LH (Peyron et al., 1998; Baldo et al., 2003), and ORX increases the activity of VP neurons through the combined activation of orexin 1 and 2 receptors (Ji et al., 2018). Dysfunction of the ORX system has been implicated in SUDs and mood disorders (Mahler et al., 2014a). The VP appears to be a critical site through which ORX regulates motivated and emotional behavioral responses, as local orexin microinfusion nearly doubles appetitive hedonic responses to sucrose in rats (Ho and Berridge, 2013). Similarly, ORX signaling in the VP regulates the motivation of rats to self-administer the opioid remifentanil and promotes relapse to remifentanil seeking (Mohammadkhani et al., 2019, 2020). These findings strongly implicate VP ORX signaling in motivated drug-seeking and hedonic processing. Conversely, pharmacological blockade or knockdown of orexin receptors in the VP produces anhedonia and behavioral despair (Ji et al., 2018).

The VP also receives moderate histamine inputs from the tuberomammillary nucleus of the hypothalamus (Panula et al., 1989), which acts through H1 and H2 receptors to promote the activation of VP_GABA_ neurons (Ji et al., 2018). Although the VP also receives noradrenergic inputs from the locus coeruleus and nucleus of the solitary tract (Delfs et al., 1998), the role of this neurotransmitter in VP has not been studied and the behavioral significance of either histamine or noradrenalin signaling in the VP remains currently unknown.

## Part 4: Role of ventral striatopallidal afferents in the regulation of motivated behaviors

### Reward processing by D1- and D2-VP projections—complementary or opposing roles

The VP has long been suspected to receive GABAergic inputs from both D1- and D2-MSNs (Heimer et al., 1991; Lu et al., 1998), and this was conclusively demonstrated in recent optogenetic studies (Kupchik et al., 2015; Creed et al., 2016; Matsui and Alvarez, 2018). D2-MSNs almost exclusively innervate the VP, whereas D1-MSNs that innervate the VP collateralize to the VTA and other structures along the medial forebrain bundle (Tripathi et al., 2010; Pardo-Garcia et al., 2019). Nevertheless, distinct populations of D1-MSNs likely exist that prominently innervate either the VP or the VTA (Baimel et al., 2019). Cell-type specific connectivity has been shown between the NAc and the VP. D1-MSNs preferentially innervate VP_Glu_ and VP_Penk_ neurons, whereas VP_GABA_ and VP_PV_ neurons receive equal innervation from D1- and D2-MSNs (Knowland et al., 2017; Heinsbroek et al., 2020).

D1- and D2-MSNs are thought to have dichotomous functions in reward processing, with D1-MSNs regulating reward and positive reinforcement and D2-MSNs mediating negative reinforcement and aversion (Hikida et al., 2010; Lobo et al., 2010; Kravitz et al., 2012; Tai et al., 2012). However, recent studies are challenging the notion that D1- and D2-MSNs oppositely regulate motivation. Optogenetic activation of either D1- or D2-MSNs promotes ICSS (Cole et al., 2018), and brief optical activation of D1- or D2-MSNs during reward-predicting cues enhances the motivation to obtain food rewards (Soares-Cunha et al., 2016b, 2018). Part of this behavioral effect is mediated by a transient decrease in the activity of VP neurons, the disinhibition of VTA_DA_ neurons (Soares-Cunha et al., 2018, 2020), and a consequent increase in motivation (Ilango et al., 2014; Mohebi et al., 2019; Ferguson et al., 2020).

The motivation-enhancing effects of D2-MSN stimulation during cue presentation are mediated by D2-VP projections. Yet if the D2-VP pathway is instead activated during reward delivery a decrease in motivation is observed (Soares-Cunha et al., 2022). Combined, these findings indicate differential engagement of the D2-VP pathway during two distinct phases of reward-seeking behavior: the motivation towards obtaining a reward that is invigorated by incentive stimuli, and the monitoring of expected outcomes by prediction error processing. Thus, the reduction in reward-seeking observed following the activation of D2-VP neurons during reward retrieval may produce a negative prediction error in the VP that reduces motivation (Soares-Cunha et al., 2022).

Interestingly, studies that use more prolonged inhibition of D2-MSNs or the D2-VP pathway show an increase in the motivation to seek rewards during a progressive ratio test without affecting response rates or reward devaluation sensitivity (Bock et al., 2013; Carvalho Poyraz et al., 2016; Gallo et al., 2018). An additional level of complexity in the D2-VP pathway has been noted by the observation that optogenetic stimulation of D2-VP neurons in the dorsomedial NAshell produces reward, while stimulation of the D2-VP pathways originating from the ventromedial or ventrolateral NAshell is aversive (Yao et al., 2021). Combined, these studies reveal a complex regulation of reward-seeking by the D2-VP pathway that is dependent on different anatomical subcircuits, the duration of the manipulation, and the phase of reward-seeking.

### D1- and D2-VP regulation of aversion—role of endogenous opioids

D1- and D2-MSNs are involved in both reward and aversion processing (Steinberg et al., 2014; Al-Hasani et al., 2015; Soares-Cunha et al., 2016b, 2018, 2020; Natsubori et al., 2017; Cole et al., 2018; Lafferty et al., 2020), which is mediated by differences in the duration of their activation (Soares-Cunha et al., 2020). Brief optogenetic stimulation of D1- or D2-MSNs produces CPP through the disinhibition of VTA_DA_ neurons, which is mediated by VTA_GABA_ neurons and the VP respectively (Soares-Cunha et al., 2020). However, prolonged, high-frequency-like stimulation of either D1- or D2-MSNs produces a conditioned place aversion (Soares-Cunha et al., 2020). Interestingly aversion following prolonged D1- or D2-MSN stimulation is mediated by opioid neurotransmission. Blocking KORs in the VTA blocks the prolonged D1-VTA stimulation-induced aversion while blocking DORs in the VP prevents the aversive effects of prolonged D2-VP stimulation (Soares-Cunha et al., 2020). The regulation of reward and aversion by D1-MSNs is also mediated by their differential projections to the VP and VTA. D1-VP projections are activated by aversive stimuli, whereas D1-VTA projections show increased activity to rewarding stimuli. Conversely, optogenetic activation of the D1-VTA pathway or inhibition of the D1-VP pathway produces CPP, whereas stimulation of the D1-VP or inhibition of the D1-VTA pathway produces a conditioned place aversion (Liu et al., 2022). Thus, different D1-MSN projections and co-released GABA and neuropeptides from D1- and D2-MSNs produce opposite effects on behavior.

### D1- and D2-VP regulation of drug reward and drug-seeking—converging or opposing roles?

A number of studies have reported that D1-MSNs potentiate, and D2-MSNs attenuate the conditioned rewarding and behavioral sensitizing effects of drugs of abuse (Lobo et al., 2010; Ferguson et al., 2011; Chandra et al., 2013; Koo et al., 2014; Calipari et al., 2016). In addition, D2-MSNs limit cocaine reinforcement in a self-administration model (Bock et al., 2013), and D1- and D2-VP manipulations differentially affect relapse to drug-seeking. Inhibition of the combined D1/D2-MSN striatopallidal pathway reduces reinstatement of cocaine seeking (Stefanik et al., 2013a), and this effect is mediated by the D1-VP component of this pathway (Heinsbroek et al., 2017; Pardo-Garcia et al., 2019). However, chemogenetic inhibition of the combined D1-/D2-MSN striatopallidal pathway promotes heroin seeking in a subset of addiction-prone rats, which indicates that this pathway may differentially regulate relapse for different drugs of abuse (O’Neal et al., 2020).

The abovementioned studies suggest opposing roles for D1- and D2-MSNs in drug reward and drug-seeking, but brief optogenetic activation of either D1- or D2-MSNs is capable of increasing the conditioned rewarding effects of cocaine (Soares-Cunha et al., 2020). By contrast, prolonged high-frequency-like stimulation of D2-MSNs reduces the rewarding effects of cocaine (Soares-Cunha et al., 2020). The latter effect is most likely mediated by the endogenous opioid enkephalin, which is co-released in the VP from D2-MSN terminals under these conditions and produces an aversive state (Soares-Cunha et al., 2020). Cocaine reward is also differentially mediated by different D1-MSN projections. Optogenetic stimulation of the D1-VP pathway, or inhibition of the D1-VTA pathway reduces the conditioned rewarding effects of cocaine (Liu et al., 2022).

Altogether, the data gathered so far show a nuanced regulation of motivated behaviors by D1- and D2-VP projections that go beyond the simple inhibition of the VP by GABA released from these neurons, and likely involves the co-release of GABA and opioids. In addition, the differential activation of D1- and D2-MSNs by rewarding and aversive stimuli likely produces distinct behavioral responses (Soares-Cunha et al., 2020). Drugs of abuse are known to produce D1- and D2-VP specific changes in the functioning of these pathways and these findings are discussed in section “Part 6: Experience dependent changes in ventral pallidal circuit function.”

## Part 5: The regulation of motivated behavior by ventral pallidal efferents

VP neurons collateralize heavily between downstream targets (Tripathi et al., 2013; Feng et al., 2021). However, most VP cells only provide dense axonal innervation to between one and three downstream regions, and sparse collateral innervation of other areas (Tripathi et al., 2013). This may explain some discrepancies in the literature between single cell axonal reconstruction studies that show dense collateralization of VP neurons and retrograde tracing studies that overall show low levels of overlap in the innervation of multiple structures by VP neurons (Tripathi et al., 2013; Leung and Balleine, 2015; Prasad and McNally, 2016; Bernat et al., 2021). Nonetheless, major differences have been reported in the electrophysiological properties, afferent innervation, and gene expression of VP neurons based on their innervation of downstream structures (Knowland et al., 2017; Bernat et al., 2021; Pribiag et al., 2021; Engeln et al., 2022). Thus, although VP neurons heavily collateralize, they can be functionally distinguished by their main projection target.

### Ventral mesencephalon and habenular projections—regulation of the dopamine system

**Figure 5:**
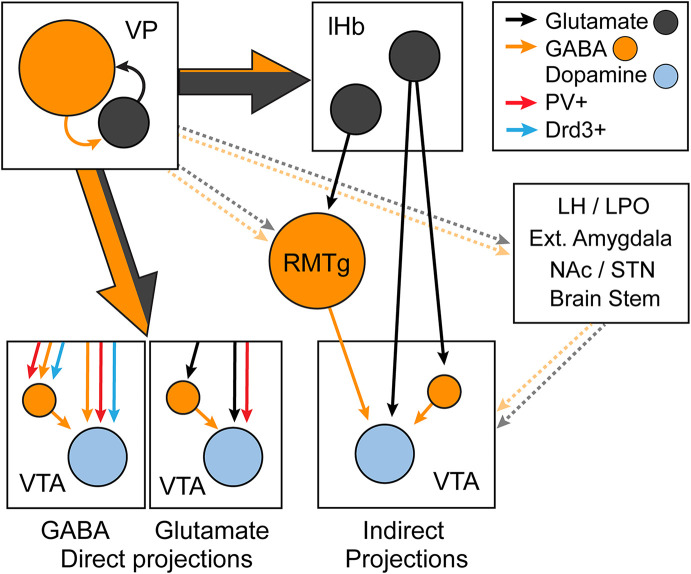
Diagram summarizing the direct and indirect regulation of ventral tegmental area (VTA) dopamine neurons by different subpopulations of VP neurons. The direct VP-VTA projection is predominantly glutamatergic, and the indirect VP to lateral habenula (lHb) to VTA projection is mostly glutamatergic. GABAergic VP-VTA projections include parvalbumin (PV) and dopamine D3 receptor (Drd3) positive projections and equally innervate VTA dopamine and GABA neurons. Glutamatergic VP-VTA neurons are either PV positive or negative, and PV-positive projections preferentially target dopamine neurons. lHb neurons that are innervated by the VP project directly to the VTA, and indirectly inhibit dopamine neurons through the rostromedial tegmentum (RMTg). Other targets through which VP neurons influence dopamine neuron activity include the lateral hypothalamus (LH) and preoptic area (LPO), extended amygdala, nucleus accumbens (NAc), and subthalamic nucleus (STN) and brain stem nuclei. Dotted lines indicate uncharacterized and hypothesized projections.

Initial characterization of the VP-VTA pathway showed that the pharmacological inhibition of the VP disinhibits VTA_DA_ population activity and promotes a tonic release of DA in the NAc (Floresco et al., 2008). However, subsequent work has shown that projections from the VP to the VTA are capable of either inhibiting or disinhibiting downstream VTA_DA_ neurons (Mahler et al., 2014b; Tooley et al., 2018; Wulff et al., 2019). VTA_DA_, VTA_GABA_, and glutamatergic VTA (VTA_Glu_) neurons receive a similar proportion of monosynaptic inputs from the VP compared to other regions (Watabe-Uchida et al., 2012; Beier et al., 2015, 2019; Faget et al., 2016), but preferential connectivity between subpopulations of VP and VTA neurons has been reported. VP_GABA_ (and GABAergic VP_PV_ neurons) provide equal inhibitory inputs to VTA_DA_ and VTA_GABA_ neurons (Knowland et al., 2017; Soden et al., 2020), and the mostly GABAergic VP_Drd3_ population equally innervates VTA_DA_, VTA_GABA_, and VTA_Glu_ neurons (Pribiag et al., 2021). By contrast, VP_Glu_ neurons (at least those that express PV) preferentially innervate VTA_DA_ neurons (Knowland et al., 2017). The VP also regulates VTA_DA_ indirectly through VP_GABA_ and VP_Glu_ projections to the lHb and RMTg (Yetnikoff et al., 2015; Zahm, 2016; Faget et al., 2018; Stephenson-Jones et al., 2020). In addition, the VP may indirectly regulate VTA_DA_ neuron activity through other regions (e.g., the LH, lateral preoptic area, extended amygdala, and pedunculopontine tegmentum) (Watabe-Uchida et al., 2017). Thus, VP_GABA_ and VP_Glu_ are capable of either inhibiting or disinhibiting VTA_DA_ neurons through multiple circuit mechanisms, which are summarized in Figure 5.

Behavioral studies largely confirm a rewarding and behaviorally reinforcing role for VP_GABA_-VTA projections, and an aversive role for VP_Glu_-VTA projections (Faget et al., 2018). VP_GABA_-VTA projections are critically involved in cocaine and alcohol seeking after extinction (Mahler and Aston-Jones, 2012; Mahler et al., 2014b; Prasad et al., 2020), whereas VP_Glu_-VTA projections might not be involved in relapse to cocaine-seeking (Mahler et al., 2014b). The mixed GABAergic and glutamatergic VP_PV_-VTA projection also drive relapse to alcohol seeking after extinction (Prasad et al., 2020). Interestingly, while activity in the mostly GABAergic VP_Drd3_-VTA projection was shown to be rewarding, and to increase DA in the NAc, chemogenetic inhibition of this pathway potentiates cocaine seeking after abstinence, which indicates a relapse-limiting role for this projection (Pribiag et al., 2021). In line with this finding, inhibiting VP-VTA projections potentiates the expression of cocaine CPP after abstinence (Bernat et al., 2021). Combined, these studies paint a complex picture whereby the VP-VTA pathway can either act as a driver or limiter of drug-seeking behavior dependent on whether extinction has occurred. The VP_PV_-VTA pathway also regulates social reward, and neuroadaptations in this pathway promote social avoidance after social defeat stress (Knowland et al., 2017).

Although the VP-lHb pathway is predominantly glutamatergic (Barker et al., 2017; Knowland et al., 2017; Faget et al., 2018), opposing behavioral effects are observed following the manipulation of VP_Glu_ and VP_GABA_ projections to the lHb. Optogenetic stimulation of VP_GABA_-lHb projections is not acutely rewarding, but inhibiting this pathway disrupts positive and negative reinforcement and reward-seeking behavior (Faget et al., 2018; Stephenson-Jones et al., 2020). Conversely, stimulating the predominantly GABAergic VP_Drd3_-lHb projection is rewarding and promotes DA release in the NAc. VP_Drd3_-lHb projections also promote cocaine seeking after abstinence, and this behavior can be blocked by the selective ablation of the dopamine D3 receptor in this pathway (Pribiag et al., 2021). However, despite the involvement of the VP-lHb projection in reward-seeking, this projection does not appear to regulate alcohol consumption (Sheth et al., 2017).

Optogenetic stimulation of VP_Glu_-lHb projections is aversive, and the inhibition of this pathway disrupts negative reinforcement (Faget et al., 2018; Stephenson-Jones et al., 2020). In depression models, stimulating VP_Glu_-lHb projections during social defeat stress exacerbates behavioral despair and a loss of interest in social reward, while inhibiting this projection during social defeat confers protection against the development of a depressive-like state (Liu et al., 2020). Stimulating the predominantly glutamatergic VP_PV_-lHb pathway also exacerbates behavioral despair after social defeat stress, while despair is reduced by inhibiting this pathway (Knowland et al., 2017). Although these behaviors are likely mediated in large part by the negative regulation of the DA system by the lHb, VP neurons also contact lHb neurons that project to the dorsal raphe and thus, the 5HT system may be implicated in these behaviors as well (Stephenson-Jones et al., 2020).

### Lateral hypothalamus and subthalamic projections

Of all VP neurons segregated by their projection targets, VP-LH neurons show the least collateralization to other regions and can be distinguished by a unique gene expression profile and a higher intrinsic excitability compared to other VP projection neurons (Bernat et al., 2021). The VP-LH pathway drives cocaine seeking in a CPP model, and both VP_GABA_ projections to the LH drive relapse to alcohol seeking (Prasad et al., 2020; Bernat et al., 2021). In addition, the LH is required for compulsive feeding behavior elicited by the pharmacological disinhibition of the VP with GABA antagonists (Stratford and Wirtshafter, 2012). Combined these data strongly indicate that the VP-LH pathway is a critical regulator of motivated behaviors. Both VP_Glu_ and VP_GABA_ densely innervate the LH (Faget et al., 2018), and motivated behaviors are differentially regulated in the LH by ORX, GABA, and glutamate neurons (Stuber and Wise, 2016). Thus, elucidating the connectivity between subpopulations of VP and LH neurons will provide important insight into the regulation of reward and motivation.

Based on the preferential inputs from the dlVP to the STN, interrogating VP-STN projections could provide useful insight into the role of the dlVP transpallidal circuit (Zahm and Heimer, 1990). Although the dlVP-STN projection has long been speculated to regulate motor functions (Zahm, 1999), inhibition of dlVP-STN neurons does not alter amphetamine-induced locomotor activity or psychomotor sensitization (Nakata et al., 2022). However, dlVP-STN projections are implicated in relapse to alcohol seeking, and a conditioned sensitized motor response to repeated amphetamine injections (Prasad and McNally, 2016; Nakata et al., 2022). Thus, dlVP-STN projections relay motivational information.

### Pallidothalamic projections

VP projections to the MD are critically important for reward learning. Interfering with the functioning of a NAshell-vmVP-MD circuit blocks the capacity of incentive conditioned stimuli to drive reward-seeking during Pavlovian-instrumental transfer (Leung and Balleine, 2013, 2015). Furthermore, genetic knockout of the Nuclear receptor family 4a1 transcription factor (Nr4a1) in VP-MD projections disrupts the acquisition of cocaine self-administration (Engeln et al., 2022). However, while the VP-MD projection is critical for new reward learning, established reward-seeking behaviors may not require activity in the VP-MD circuit, since pharmacological inhibition of the MD does not impact relapse to cocaine-seeking (McFarland and Kalivas, 2001). These findings match observations where MD lesions impair reward learning but do not affect reward-seeking in well-trained animals unless changes in strategy are required to solve a task (Mitchell and Chakraborty, 2013). In addition to reward learning, VP-MD projections are involved in the regulation of exploratory locomotor activity (Churchill and Kalivas, 1999), and attention and cognition. For instance, pharmacological VP-MD perturbations have been shown to disrupt working memory in rats (Kalivas et al., 1999, 2001).

### Pallidostriatal projections

Reciprocal connections between the NA shell and VP are required for the experience of positive hedonic states (Smith and Berridge, 2007), and stimulation of the “arkypallidal” VP-NA shell projection increases the hedonic value or “liking” of sucrose reward (Vachez et al., 2021). VP-NAshell projections are GABAergic and cholinergic, and innervate both D1- and D2-MSNs and a small population of striatal interneurons (Churchill and Kalivas, 1994; Faget et al., 2018; Li et al., 2018; Vachez et al., 2021). Reward consumption activates VP-NAshell neurons, and stronger activation of this pathway predicts a longer duration of reward consumption. Interestingly, arkypallidal VP-NAcore projections do not regulate relapse to cocaine-seeking (Stefanik et al., 2013a). One potential explanation for this finding is that arkypallidal VP projections may exclusively signal the hedonic value or “liking” of reward, and not the incentive motivation or “wanting” of rewards that would be expected to drive craving and relapse to cocaine seeking. Indeed, stimulating arkypallidal VP neurons is not rewarding or reinforcing by itself (Vachez et al., 2021). Differences in the connectivity of arkypallidal VP neurons have been reported between species. In the mouse brain pallidostriatal cells provide relatively specific projections to the NAc, but in the rat brain these neurons send dense collaterals to the thalamus and brain stem (Tripathi et al., 2013; Vachez et al., 2021).

## Part 6: Experience dependent changes in ventral pallidal circuit function

### Drug-induced changes in ventral pallidal function

Drug exposure produces pronounced changes in ventral striatopallidal circuit function. Cocaine produces a gain of function in D1-VP neurons that is measured as a postsynaptic strengthening of glutamatergic neurotransmission onto these cells (Baimel et al., 2019; Inbar et al., 2022) and a potentiation of GABAergic neurotransmission in D1-VP synapses (Creed et al., 2016). Cocaine also potentiates glutamate release onto D2-VP cells through a presynaptic mechanism but produces a deficit in GABAergic neurotransmission in D2-VP synapses (Kupchik et al., 2014; Creed et al., 2016; Heinsbroek et al., 2017).

Chronic cocaine exposure, or cocaine self-administration also alters the ability of enkephalin to regulate striatopallidal synaptic transmission. Cocaine produces a reduction in GABA transmission in the VP and the loss of a presynaptic long-term depression (LTD) of D2-VP synapses that is mediated by an enhanced enkephalin tone on MORs and DORs (Kupchik et al., 2014; Creed et al., 2016; Heinsbroek et al., 2017). Interestingly, reversing the loss of LTD at this synapse using an optogenetic *in vivo* long-term potentiation (LTP) protocol reduces cocaine-withdrawal-induced motivational deficits and negative affect measured by reduced sucrose “wanting” and “liking” (Creed et al., 2016). In contrast to D2-VP projections, D1-VP projections show a loss of LTP following cocaine exposure which is mediated by a persistent potentiation of this synapse. LTP in D1-VP synapses is mediated by a dopamine D1 receptor-mediated activation of protein kinase A, and reversing this plasticity with an *in vivo* optogenetic LTD protocol abolished behavioral sensitization to cocaine (Creed et al., 2016). Combined, these data show that a cocaine-induced strengthening of the D1-VP pathway mediates sensitized behavioral responses and promotes relapse to cocaine-seeking (Creed et al., 2016; Pardo-Garcia et al., 2019). Meanwhile, an Enkephalin-mediated loss of function in the D2-VP pathway produces withdrawal-induced negative affect and reduces inhibitory control over cocaine seeking (Creed et al., 2016; Heinsbroek et al., 2017; Soares-Cunha et al., 2020). In addition, cocaine withdrawal produces a synaptic disinhibition of VP_Glu_ neurons (Inbar et al., 2020), and VP_Glu_ neurons show increased activity during cocaine withdrawal after extinction training (Heinsbroek et al., 2020, Cell Reports). However, the precise contributions of VP_Glu_ neurons to drug withdrawal requires further investigation. In addition, whether the same circuit adaptations are produced by drugs other than cocaine remains to be determined.

Chronic cocaine use increases dynorphin production in D1-MSNs, and dynorphin concentrations are increased in both the NAc and VP in human cocaine users (Carlezon et al., 1998; Frankel et al., 2008). Cocaine potentiates the ability of dynorphin to inhibit synaptic transmission onto VP_GABA_ neurons and abolishes the capacity of dynorphin to potentiate GABA transmission onto VP_Glu_ neurons. The resulting dynorphin-mediated disinhibition of the D1-VP pathway may promote relapse to cocaine-seeking (Pardo-Garcia et al., 2019), while the disinhibition of VP_Glu_ may mediate a state of withdrawal-induced negative affect (Inbar et al., 2020).

An increase in VP_Glu_ synaptic strength onto lHb neurons is also observed after cocaine withdrawal, which is measured by a postsynaptic increase in the ionotropic glutamate AMPA/NMDA receptor ratio and a presynaptic increased neurotransmitter release probability in this synapse. By contrast, VP_Glu_ synapses onto neighboring VP neurons, and synapses onto VTA_DA_ and VTA_GABA_ neurons are weakened (reduced AMPA/NMDA ratio) after cocaine withdrawal, although an increased release probability is observed in VP_Glu_-VTA_GABA_ synapses (Levi et al., 2020). These complex circuit-specific VP_Glu_ neuroadaptations may mediate withdrawal-induced negative affect, but this requires further investigation.

In addition to synaptic changes, drug exposure also produces cell- and pathway-specific changes in the intrinsic excitability and transcriptome of VP neurons (Pribiag et al., 2021; Engeln et al., 2022). Cocaine self-administration alters the expression of a number of genes implicated in neuronal excitability, neurotransmission, and functional and structural plasticity that share regulation by Nr4a1. Nr4a1 is selectively upregulated in VP-MD projections following cocaine self-administration, and overexpression of Nr4a1 increases cued and cocaine-primed cocaine relapse. Interestingly, pathway-specific VP-MD knock-out of Nr4a1 blocked the acquisition of cocaine self-administration, which supports the notion that VP-MD projections are crucial for reward learning. Cocaine also increased spine density on VP-MD neurons, an effect that was blocked by Nr4a1 overexpression (Engeln et al., 2022). These data provide the first exciting indication that drug exposure alters structural glutamatergic synaptic plasticity in the VP and match earlier reports that cocaine increases glutamatergic vs. GABAergic neurotransmission in the VP (McDaid et al., 2006; Pribiag et al., 2021).

### Stress-induced changes in ventral pallidal function

Stressful life experiences produce pronounced changes in VP function, and stress is a major contributing factor to the development of mood disorders and SUDs (Knoll and Carlezon, 2010; Kwako and Koob, 2017; Levis et al., 2022). Thus, understanding changes produced by stress on VP reward processing has important implications for our understanding of these disorders.

Chronic unpredictable mild stress increases the inhibition of VTA_DA_ neurons by the VP, an effect that is mediated by increased glutamatergic neurotransmission in the VP originating from the BLA (Chang and Grace, 2014). Chronic social defeat stress also increases glutamatergic neurotransmission onto VP_PV_-VTA neurons while simultaneously reducing GABAergic neurotransmission onto VP_PV_-lHb neurons (Knowland et al., 2017). In animals susceptible to chronic social defeat stress, both excitatory and inhibitory transmission are reduced onto VP neurons. However, inhibition is altered more than excitation, which results in an increased excitation over inhibition ratio recorded from individual cells (He et al., 2020). Collectively, these studies indicate that chronic stress rearranges synaptic inputs in the VP and leads to a relative increase in excitatory over inhibitory neurotransmission in the VP.

In addition, chronic stress alters the intrinsic excitability of VP neurons. VP_PV_-lHb neurons show an increase in excitability following stress, whereas a reduction in the excitability of VP_PV_-VTA neurons is associated with resilience against a stress-induced depression-like state (Knowland et al., 2017). Other work indicates that social defeat stress reduces the overall excitability of VP neurons and that these effects are mediated by a reduced release of substance P in the VP from the NAc (He et al., 2020). In support of this, this study showed that the blockade of VP neurokinin 1 receptor produces a depressive-like state characterized by social withdrawal, anxiety, and reduced sucrose preference (He et al., 2020). Importantly, both stress-induced changes in the synaptic excitation over inhibition ratio and the intrinsic excitability in the VP can be reversed by chronic antidepressant treatment (Knowland et al., 2017).

Chronic restraint stress also changes the function of striatopallidal projections, and upregulates Pituitary Adenylate Cyclase-Activating Polypeptide (PACAP) production in a ventrolateral NAshell to vlVP circuit. In the VP the Pacap 1 receptor is predominantly expressed on VP_GABA_ neurons and viral knockdown of this receptor or chemogenetic inhibition of the lateral NAshell-VP projection reduces behavioral despair after chronic restraint stress. Interestingly, home cage enrichment or antidepressant treatment normalizes PACAP signaling in this circuit and prevents the development of a depressive-like state (Park et al., 2022). Collectively, these findings indicate that chronic stress produces neuroadaptations in the VP that impair reward processing and produce negative affect.

## Part 7: Summary and concluding remarks

The literature summarized here shows an emerging central role for the VP in the regulation of motivated behavior, hedonic states, reinforcement, and reward and aversion processing. Arguably the most important discoveries of the last decade are that VP_GABA_ and VP_Glu_ oppositely regulate motivation and reinforcement and that the VP receives dense functional innervation from both NAc D1- and D2-MSNs. Although these findings have greatly increased our understanding of reward processing by the VP, many questions remain. For instance, precisely how differential connectivity between NAc and VP neurons contributes to reward processing is largely unclear. Furthermore, whether differences in opioid and neuropeptide modulation of striatopallidal circuits influences behavioral outcomes following stressful experiences or exposure to drugs of abuse requires further examination.

Our understanding of the cellular heterogeneity in the VP is rapidly advancing with the use of novel genetic approaches and by interrogating the roles of different VP neurons using opto- and chemogenetics. However, we are likely only at the early stages of a full understanding of the contributions of different projection- and genetically defined neuronal populations in the VP to motivated states. The coming years will no doubt bring more exciting insights into the roles of distinct VP populations, subregions, and circuits in motivation and reward processing. Although distinct VP subregions were discovered decades ago (Zahm and Heimer, 1990), the precise role of these regions and connected circuits in motivated behaviors still requires additional clarification. Research is beginning to define differences in circuit function and cellular subcomposition of the VP along the rostrocaudal axis (Kupchik and Kalivas, 2013; Mahler et al., 2014b; Faget et al., 2018; Tooley et al., 2018), and the use of similar genetic interrogation, tracing and electrophysiological approaches to the dorsolateral, ventromedial and ventrolateral VP may provide tremendous insights into the contributions of these VP subregions to motivated behaviors.

Overall, nearly five decades of research into the VP has led to a profound understanding of the role of this structure in reward processing and motivation, and the disruption of these functions in neuropsychiatric disorders. The culmination of this research may one day lead to the selective targeting of the VP for the treatment of psychiatric conditions. Of note, ischemic lesions in the ventral anterior globus pallidus in humans have been reported to produce a sustained remission of SUD, and deep brain stimulation near this region reduces impulsivity (Moussawi et al., 2016, 2022). These effects do however seem to require precise targeting, as lesions or electrical stimulation in adjacent pallidal areas may worsen impulsivity and depression symptoms (Miller et al., 2006; Moussawi et al., 2022). Nonetheless, specifically tuned electrical deep brain stimulation of the VP may have therapeutic benefits. In addition, future more selective chemogenetic or optogenetic manipulations of the VP may one day provide an effective treatment for neuropsychiatric conditions.

## Author contributions

JH and CS-C wrote the manuscript. All authors contributed to the article and approved the submitted version.

## Figures and Tables

**Table:** 

5HT	Serotonin
ACh	Acetylcholine
BLA	Basolateral amygdala
CPP	Conditioned place preference
D1	Dopamine D1 receptor
D1-MSN	Dopamine D1 receptor-expressing medum spiny neuron
D2	Dopamine D2 receptor
D2-MSN	Dopamine D2 receptor-expressing medum spiny neuron
DA	Dopamine
dIVP	Dorsolateral ventral pallidum
DOR	Delta opioid receptor
EPM	Elevated plus maze
GAT-3	GABA transporter 3
GP	Globus pallidus
ICSS	Intracranial self-stimulation
KOR	Kappa opioid receptor
LH	Lateral hypothalamus
lHb	Lateral habenula
LTD	Long-term depression
LTP	Long-term potentiation
MD	Mediodorsal thalamus
MOR	Mu opioid receptor
MSN	Medum spiny neuron
NAc	Nucleus accumbens
NAcore	Nucleus accumbens core
NAshell	Nucleus accumbens shell
Npas1	Neuronal Per-Arnt-Sim domain 1
Nr4a1	Nuclear receptor family 4a1 transcription factor
OF	Open field
ORX	Orexin
OT	Olfactory tubercle
PACAP	Pituitary Adenylate Cyclase-Activating Polypeptide
Penk	Pro-enkephalin
PPN	Pedunculopontine nucleus
PR	Progressive ratio
PV	Parvalbumin
RMTg	Rostromedial tegmentum
RPE	Reward prediction error
RTPP	Real-time place preference
tVP	Tubercular ventral pallidum
STN	Subthalamic nucleus
SUD	Substance use disorder
Vglut2	Vesicular glutamate transporter 2
Vglut3	Vesicular glutamate transporter 3
vlV	Ventrolateral ventral pallidum
vmVP	Ventromedial ventral pallidum
VP	Ventral pallidum
VTA	Ventral tegmental area
